# Simulation of Dilated Heart Failure with Continuous Flow Circulatory Support

**DOI:** 10.1371/journal.pone.0085234

**Published:** 2014-01-17

**Authors:** Yajuan Wang, Natasha Loghmanpour, Stijn Vandenberghe, Antonio Ferreira, Bradley Keller, John Gorcsan, James Antaki

**Affiliations:** 1 Department of Biomedical Engineering, Carnegie Mellon University, Pittsburgh, Pennsylvania, United States of America; 2 University of Bern, Bern, Switzerland; 3 Mathematics, Universidade Federal do Maranhão, Maranhão, Brazil; 4 Cardiovascular Innovation Institute, University of Louisville, Louisville, Kentucky, United States of America; 5 Heart and Vascular Institute, University of Pittsburgh Medical Center, Pittsburgh, Pennsylvania, United States of America; Scuola Superiore Sant'Anna, Italy

## Abstract

Lumped parameter models have been employed for decades to simulate important hemodynamic couplings between a left ventricular assist device (LVAD) and the native circulation. However, these studies seldom consider the pathological descending limb of the Frank-Starling response of the overloaded ventricle. This study introduces a dilated heart failure model featuring a unimodal end systolic pressure-volume relationship (ESPVR) to address this critical shortcoming. The resulting hemodynamic response to mechanical circulatory support are illustrated through numerical simulations of a rotodynamic, continuous flow ventricular assist device (cfVAD) coupled to systemic and pulmonary circulations with baroreflex control. The model further incorporated septal interaction to capture the influence of left ventricular (LV) unloading on right ventricular function. Four heart failure conditions were simulated (LV and bi-ventricular failure with/without pulmonary hypertension) in addition to normal baseline. Several metrics of LV function, including cardiac output and stroke work, exhibited a unimodal response whereby initial unloading improved function, and further unloading depleted preload reserve thereby reducing ventricular output. The concept of *extremal loading* was introduced to reflect the loading condition in which the intrinsic LV stroke work is maximized. Simulation of bi-ventricular failure with pulmonary hypertension revealed inadequacy of LV support alone. These simulations motivate the implementation of an extremum tracking feedback controller to potentially optimize ventricular recovery.

## Introduction

**Figure 1:**
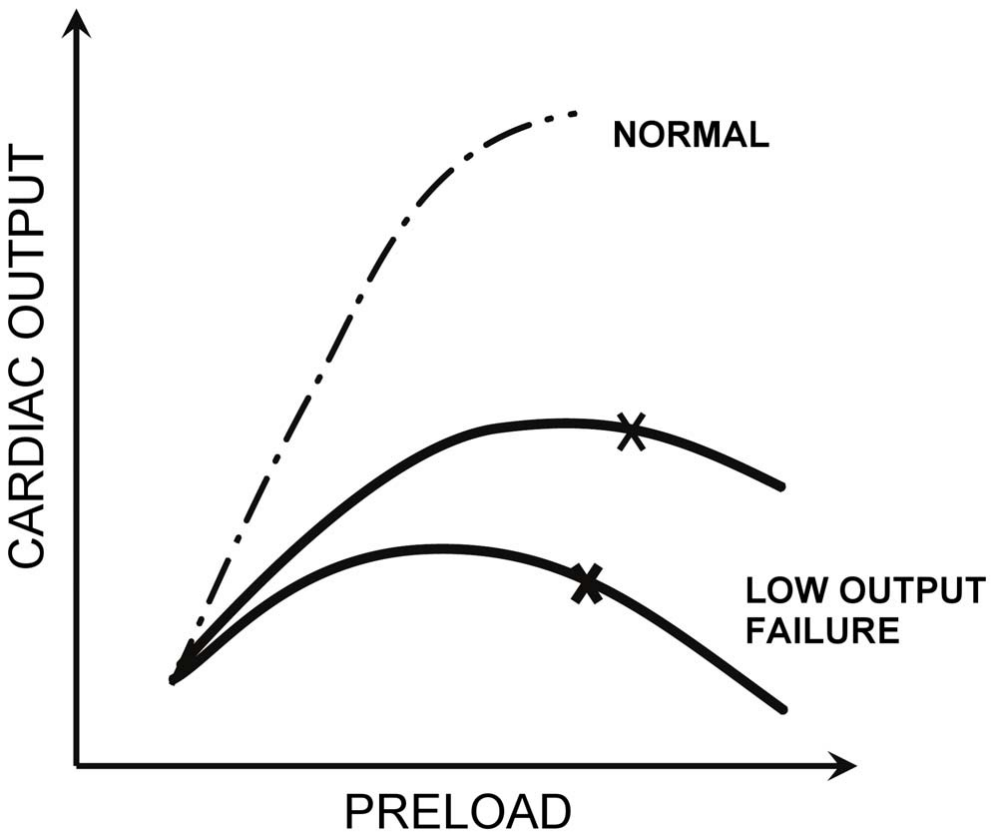
Illustration of the descending limb of the Starling curve for dilated heart failure. Adapted from McMichael.[43]

Lumped parameter models of mechanical circulatory support have been employed for the past two decades to simulate important hemodynamic couplings between a ventricular assist pump and the native circulation. In the modern era of continuous flow ventricular assist devices (cfVADs), such models have been used to identify algorithms for feedback control so as to adequately unload the native ventricle [1]–[4], avoid suction [5], and potentially promote cardiac recovery [6], [7]. Surprisingly, only a few of these simulations [2] have employed models of the diseased heart that reflect cardiac overload (myocardial overstretch)—one of the most common characteristics of end stage heart failure. Instead, the common model of cardiac dysfunction has been to attenuate ventricular contractility of a healthy heart model, defined by the classic elastance function of Suga and Sagawa [8], by reducing the value of maximal elastance (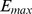) or shifting the ESPVR to the right [3], [5], [9]. This approach presumes a *monotonically increasing*, usually linear, ESPVR. But this neglects the pathological descending limb of the Frank-Starling response of the overloaded ventricle—beyond the limit of contractile reserve [10], [11] (See Fig. 1.) Consequently, these previous simulations of mechanical circulatory support unrealistically exhibit supra-physiologic cardiac contribution with increased preload, as contrasted with the failing ventricle in which excessive preload causes a depression in ejection fraction and stroke work [12], especially in acute non-ischemic cardiomyopathy.

The current study therefore investigates the hemodynamics of mechanical circulatory support with a more accurate model of the failing heart, in which the ESPVR is a unimodal function. This model provides sufficient and necessary conditions for the existence of a finite, maximum cardiac power output. A lumped parameter simulation of the systemic and pulmonary circulation with baroreflex control was implemented numerically, encompassing a variety of cardiac pathologies, including combinations of LV and right ventricular (RV) failure, with and without pulmonary hypertension. The model further incorporates right-left ventricular interdependence through the inclusion of an intraventricular septum element: to capture the influence of mechanical coupling of the two ventricles through a shared septal wall [13]. This model enables the investigation of the well-known leftward shift of the septum due to LV decompression. This effect is particularly prominent with modern cfVADs which are capable of unloading the LV to the point of suck-down. In the context of feedback control for mechanical circulatory support, this simulation study serves as a platform to evaluate extremum-tracking control [14], [15] as a potential means to optimize ventricular recovery.

## Materials and Methods

### Cardiovascular Model

**Figure 2:**
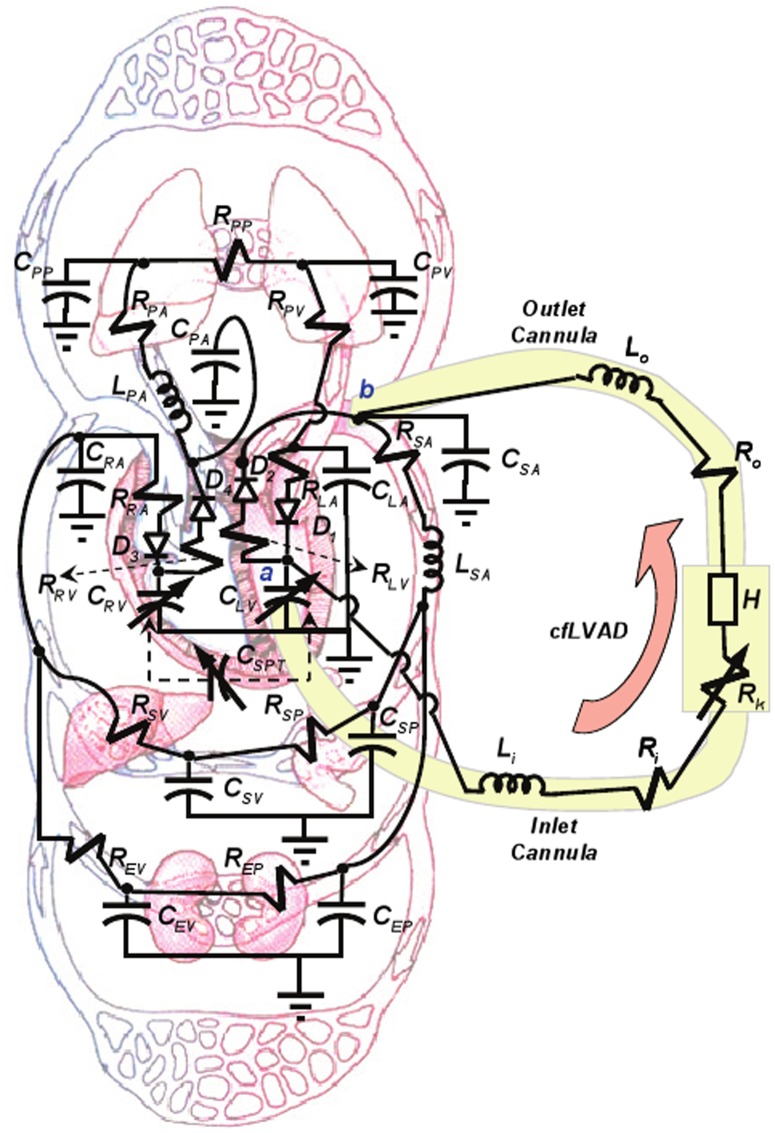
Compartmental lumped-parameter electrical analog of the circulatory system and continuous flow LVAD (cfLVAD).

A nonlinear time-varying lumped parameter model of the circulatory system, described previously by Ursino et al. [16] was modified to include an intraventricular septum element and a rotodynamic, continuous flow VAD (cfVAD). (See Fig. 2.) Elements of the vascular system were simulated using electrical analogs, wherein flow is represented by current and pressure by voltage. Passive components, namely resistors, capacitors and inductors were used to represent hemodynamic resistance, compliance and inertance, respectively [17]. This cardiovascular model consisted of the following major components: the numerical representation of left and right heart; the septum; the systemic and pulmonary vascular impedances. (See Appendix S1 for details.) Regulation of arterial pressure was achieved by a baroreflex model described previously [16].

Instantaneous ventricular pressure 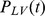 was comprised of the superposition of systolic and diastolic contributions. (See Equation (1).) The systolic component was in turn the product of a normalized time-varying activation function 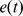 (2) and the unimodal ESPVR envelope given by (3). For the sake of simplicity, the ESPVR was idealized by a piecewise bi-linear function. The corresponding slopes and intercepts were derived from published clinical data [18]. The end-diastolic pressure-volume relationship (EDPVR) was defined by the classical exponential function given in (4). The pressure loss due to viscous dissipation within the ventricle was reflected in intraventricular resistance 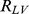 (Equation 1) as defined by Shroff et al. [19].
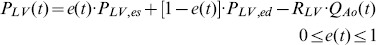
(1)

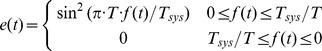
(2)where
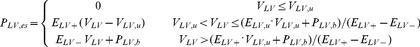
(3)and,



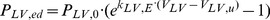
(4)

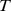
, above, is the period of the cardiac cycle, 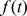 is the time fraction of cardiac cycle (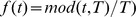, 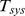 is the duration of cardiac systole; 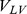 is the instantaneous LV volume, 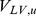 is the unstressed LV volume; 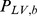 is the pressure axis intercept of the descending ESPVR. 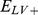, 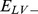 describe the slopes of piecewise ascending and descending limbs of simplified nonlinear ESPVR, respectively [19]; 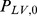 and 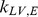 are two parameters that define the end-diastolic pressure-volume relation (EDPVR); and 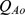 is the blood flow ejected through the aortic valve. A similar strategy was utilized to simulate the RV, with corresponding parameters (provided in the Appendix S1.) The atria were not explicitly modeled, but incorporated into respective venous reservoirs. The four heart valves were modeled as ideal diodes: 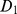 - mitral, 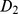 - aortic, 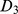 - tricuspid, 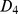 - pulmonary. By application of Kirchoffs laws, a set of thirteen state equations were derived for the full model, detailed in the Appendix S1.

To simulate ventricular interdependence, mechanical coupling between the right and left ventricles through the interventricular septum was characterized by an interventricular elastance model developed by Chung et al. [20]. The LV and RV volumes were decomposed into two parts: the neutral volumes (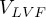 and 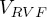) corresponding to zero inter-ventricular (transmural) pressure, and the septal displaced volume 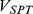 caused by the shift of the septum from neutral position towards the RV. Hence:
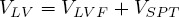
(5)

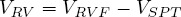
(6)where 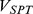 is governed by the activation function 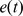 as follows:
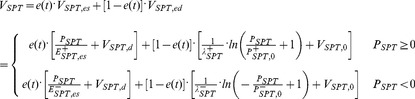
(7)where, 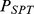 is the pressure difference across the septum:
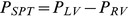
(8)


### The LVAD Model

A model of a rotodynamic, continuous flow LVAD [17] was connected to the nodes a and b of the cardiovascular model in Fig. 2, representing ventricular to aortic cannulation. It adds one state variable to the original mathematical model, namely the pressure difference across the pump H as a function of VAD flow and speed:
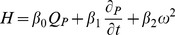
(9)where 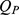 is the volumetric flow rate through the pump, 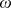 is the rotational speed, and 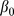, 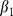, 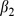 are constants that define the resistance, inertance, and speed dependence of the pump, respectively. For the purpose of the present study, parameters from a clinically used axial flow pump (HeartMate II, Thoratec, Pleasanton, CA) were implemented (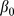 = 0.1707, 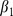 = 0.02177, 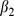 = −0.0000903) in addition to corresponding published parameters for the resistance and inertances of the inflow and outflow cannulae (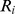 = 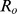 = 0.0677 mmHg*s/mL; 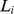 = 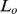 = 0.0127 mmHg*s/mL) [21].

To simulate potential suction events caused by negative pressure within the LV, an additional nonlinear suction resistor 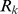 introduced by Choi was interposed between the inflow cannula and LV [21]:
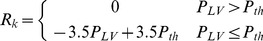
(10)where 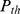 is the threshold pressure, taken as 1 mmHg.

### Simulation Protocol

A series of simulated experiments was conducted under normal conditions (no LV/RV failure) and four different pathological conditions [3], [22]–[25]: (1) left ventricular failure (LVF), (2) biventricular failure (Bi-F), (3) LVF with pulmonary hypertension (PVR+), and (4) Bi-F with PVR+. The parameter values and hemodynamic variables derived from computations under these conditions were derived from the literature and are detailed in Tables AII-AIV of Appendix S1. Hemodynamic baseline conditions of LVF with/without PVR+ were calibrated with the pre-operative conditions before LVAD implantation; similarly, baseline hemodynamics of Bi-F with/without PVR+ with those before BiVAD implantation [26], [27]. Calibration variables included systolic/diastolic/mean arterial pressure (SBP/DBP/MAP), left atrial pressure (LAP); right atrial pressure (RAP), systolic/diastolic/mean pulmonary arterial pressure (PAs/PAd/PAm), pulmonary capillary wedge pressure (PCWP), transpulmonary gradient (TPG), pulmonary vascular resistance (PVR), cardiac output (CO), and left/right ventricular ejection fraction (LVEF/RVEF).

The effect of the cfLVAD on the hemodynamics was evaluated in each of the five baseline conditions defined above. LVAD speed was increased from 6k RPM to 12k RPM in increments of 1k RPM and ventricular pressure-volume (PV) loops were composed for each, from which the following indices were evaluated: total CO (sum of aortic valve flow and LVAD flow), LAP, RAP, LV stroke volume (SV = 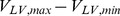 in one cardiac cycle), stroke work (SW), pre-load adjusted maximal power, fractional volume change and 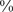 change of SV in the simulated scenario with LVAD compared to the one at the corresponding baseline. The simulation results were compared with data obtained from literature and six patient records obtained from the University of Pittsburgh Medical Center (UPMC) following approval by the Instititutional Review Board of University of Pittsburgh. Written consent was given by the patients to enroll in the study and for their medical information to be stored in the hospital database and used for research. All of these six patients were initially implanted with HeartMate II cfLVAD, among which three patients were identified with cardiac function recovery on LVAD (LVAD-removal) and the other three relied on the cfLVAD to sustain their life (LVAD-dependent). The LVAD-removal group tended to be younger (37
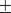
13 vs. 54
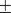
16 years); included 2 females (vs. none) and all patients suffered non-ischemic heart disease (vs. 1 in the LVAD-dependent group). The duration on LVAD in the two groups of patients when assessed were similar (93
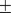
32 vs. 74
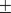
46 days in the LVAD-dependent group).

The results were further analyzed with respect to the optimality of ventricular support. Specifically, the pump speed at which the stroke work of the left ventricle was maximized was defined as *Extremal Loading*. Increasing pump speed beyond this point caused reduction of LV contribution due to reduction of preload, and decreasing speed caused LV overload.

## Results

### Simulation of Heart Failure Hemodynamics

**Figure 3:**
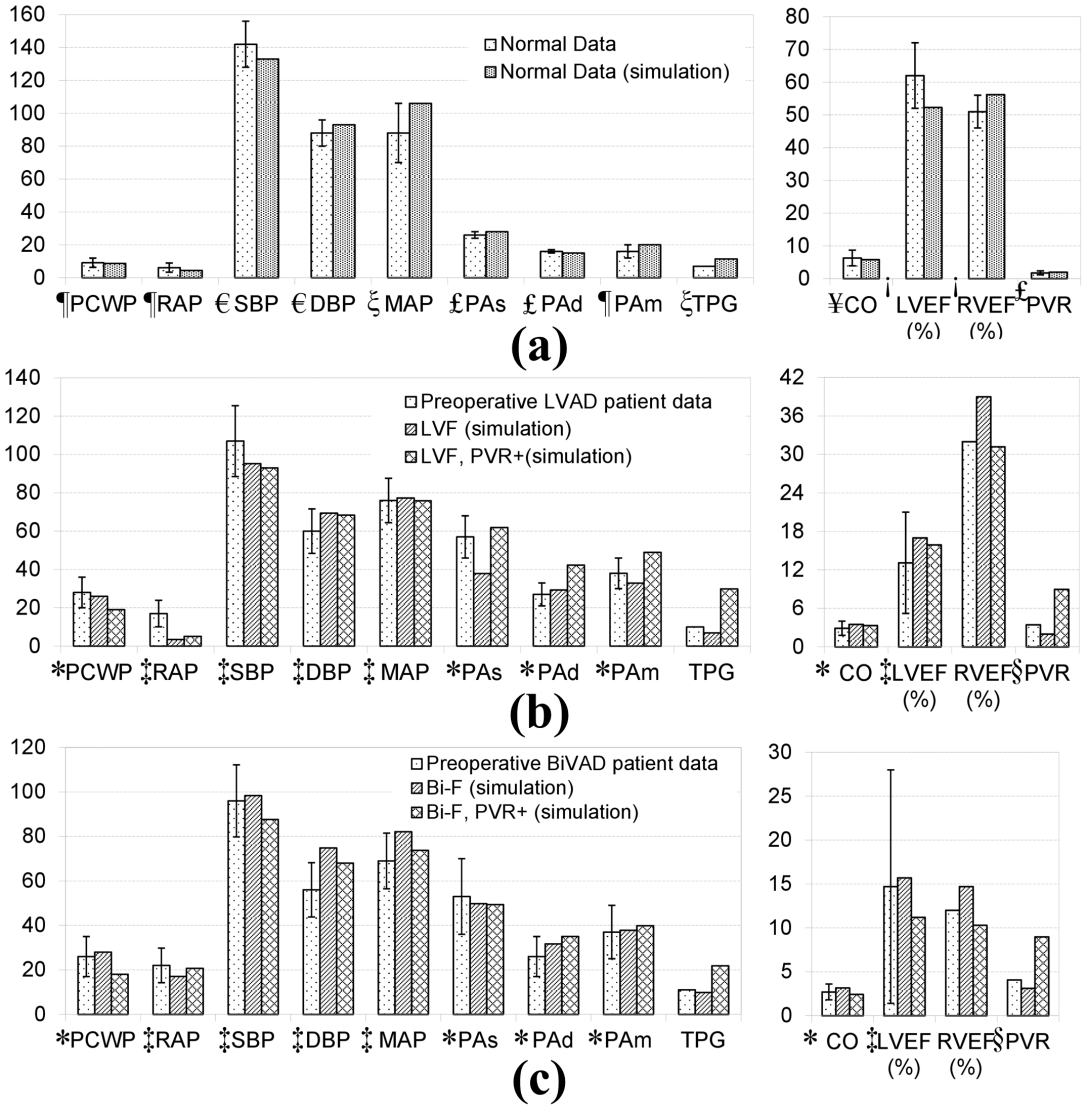
Comparison of baseline simulation data in normal and four pathological conditions with published clinical data and historical control subjects. (a) Normal patient; (b) pre-operative clinical LVAD data versus simulation baseline of LVF and LVF with PVR+; (c) pre-operative BiVAD patient data versus simulation baseline of Bi-F and Bi-F with PVR+ conditions. Values are represented as mean +/− standard deviation. (Sources: 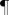 Rulli et al. 1980 [30], € Nordenfelt et al. 1985 [31], 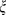 Normal range for MAP [28]£;  Ehrsam et al. 1983 [32], ! Zaret et al. 1984 [33],¥ Thadani et al. 1978 [34], * Dang et al. 2006 [26]; 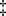 Fitzpatrick et al. 2008 [27]; 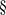 Kormos et al. [29]). All of the abbreviation are defined in Section II, Part C.

The parameters of the cardiovascular model parameters were initialized with nominal values found in the literature, and then optimized through trial-and-error, within the upper and lower ranges of normal, to approximate the primary hemodynamic variables of interest. Particular emphasis was placed on cardiac output (CO). This was performed for the normal and four heart failure pathological conditions. The corresponding hemodynamic benchmarks are provided in Table 1. Comparison to clinical values from various literature sources [26]–[34] are provided in Fig. 3. The baseline conditions are provided in Fig. 3a, illustrating a close approximation by the model to the normal adult profile. Similarly, the four simulated heart failure conditions were found to match the clinical hemodynamic data sets recorded pre-operatively in the corresponding benchmark LVAD or BiVAD data. (See Fig. 3b, c.) Although approximations were made by the lumped parameter model, most baseline values were within one standard deviation of the reported data, with the exception of PAs in LVF, RAP in LVF with/without PVR+, PAd/m in LVF with PVR+, and DBP in Bi-F without PVR+, which were within two standard deviations of the data.

**Table 1:** Simulated Hemodynamics for Normal and Four Heart Failure Conditions

Parameters	Normal	LVF	Bi-F	LVF, PVR+	Bi-F, PVR+
Cardiac output (L/min)	5.8	3.5	3.2	3.3	2.4
Stroke volume (mL)	68.0	41.1	37.1	39.2	28.6
Left atrial pressure (mean, mmHg)	8.7	26.0	28.0	19.1	18.0
Right atrial pressure (mean, mmHg)	4.4	3.5	17.1	5.1	20.7
Arterial pressure (mmHg)					
Systolic	133.0	95.2	98.4	93.0	87.6
Diastolic	93.0	69.4	74.8	68.3	68.0
Mean	106.0	77.3	82.1	75.9	73.8
Pulmonary arterial pressure (mmHg)					
Systolic	28.0	37.9	49.8	61.9	49.4
Diastolic	15.0	29.3	31.7	42.3	35.0
Mean	20.1	32.9	37.8	48.9	39.8
LV end-diastolic pressure (mmHg)	10.0	25.6	27.6	18.8	17.8
LV end-diastolic volume (mL)	130.0	245.6	237.1	246.8	256.0
RV end-diastolic pressure (mmHg)	4.0	3.3	16.8	4.8	20.5
RV end-diastolic volume (mL)	121.0	106.4	252.8	125.6	277.5
LV ejection fraction ( 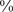 )	52	17	16	16	11
RV ejection fraction ( 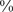 )	56	39	15	31	10
Pulmonary vascular resistance (Wood)	2.0	2.0	3.1	9.0	9.0
Heart rate (bpm)	70	85	85	85	85

See text for abbreviations.

**Figure 4:**
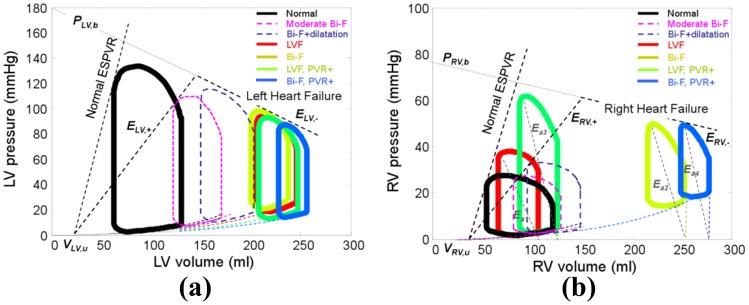
Pressure-volume relationships for normal and four heart failure conditions: (a) left ventricle; (b) right ventricle. The parameters for unimodal ESPVR in (3) are labeled in (a).

Ventricular pressure-volume (PV) loops generated for each of the simulated conditions are shown in Fig. 4, along with two intermediate conditions associated with depressed ESPVR and increased diastolic compliance. The reduction of the slope of the ascending limb of ESPVR (by 59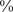) caused a characteristic depression in systolic pressure as well as a rightward shift of the PV loops due to translocation of blood to the pulmonary circuit. Increasing the diastolic compliance, corresponding to remodeling caused by volume overload, resulted in further increase of LV end-diastolic volume (EDV). This, in turn, caused the loops to enter the descending limb of the ESPVR. Similar hemodynamic effects of RV were observed in the simulated bi-ventricular failure conditions. (See Fig. 4b.) Elevation of PVR caused a more dramatic rightward shift in the RV loops than those of the LV in the conditions of both LVF and Bi-F, respectively. It also caused a commensurate increase in pulmonary effective arterial elastance (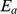), illustrated in Fig. 4b as the slope of end-systolic pressure vs. stroke volume relation [30], from 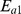 to 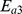, and 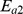 to 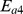, respectively.

### Hemodynamics of LVAD Unloading

**Figure 5:**
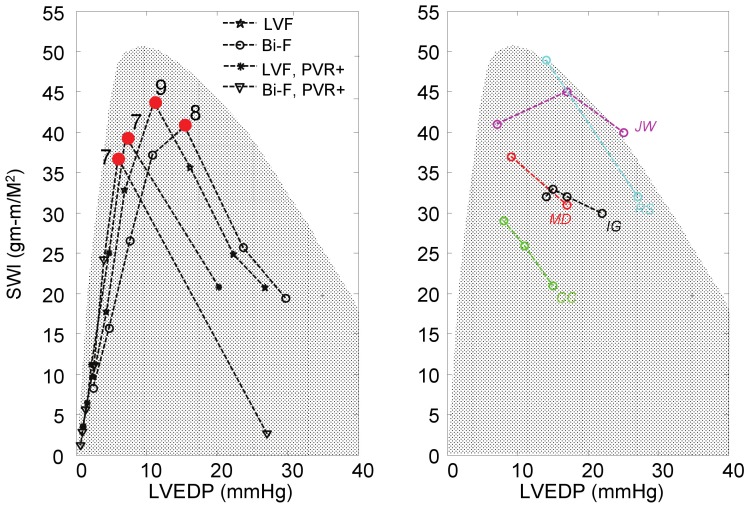
(a) Effect of varying LVAD support on LV Stroke Work Index (SWI) for four heart failure simulation cases. The red dots signify Optimal unloading, denoted with corresponding LVAD speed (kRPM). (b) Unimodal (ascending-descending) preload response resembles clinically observed severe LV dysfunction reported by Ross and Braunwald [12]. The shaded region indicates envelope of marked heart dysfunction defined by Ross and Braunwald, and based on the response of LV SWI to volume unloading in five of their patients (JW, MD, CC, IG, RS).

Fig. 5a provides the LV stroke work index (assuming 1.9 m^2^ body surface area [26]) versus end diastolic pressure (SWI-EDP) for the simulations with the cfLVAD included in the circulation. Increasing speed through the labeled operating range of the LVAD (6k–12k RPM) caused a monotonic decrease in EDP for all four simulated heart failure conditions. However, SWI exhibited a unimodal characteristic wherein the maximum was found to occur at approximately 9k RPM in LVF simulation case, 8k RPM in Bi-F, 7k RPM in LVF with PVR+ and Bi-F with PVR+, respectively. For the sake of comparison, clinically measured SWI-EDP curves of five patients with marked LV dysfunction (from Ross and Braunwald) [12] are juxtaposed (demarcated by the shaded region). (See Fig. 5b.) The latter data exhibits a similar response to preload, and illustrates a favorable response to unloading. This unimodal response is contrary to the prevailing model of a linear or monotonically increasing ESPVR.

**Figure 6:**
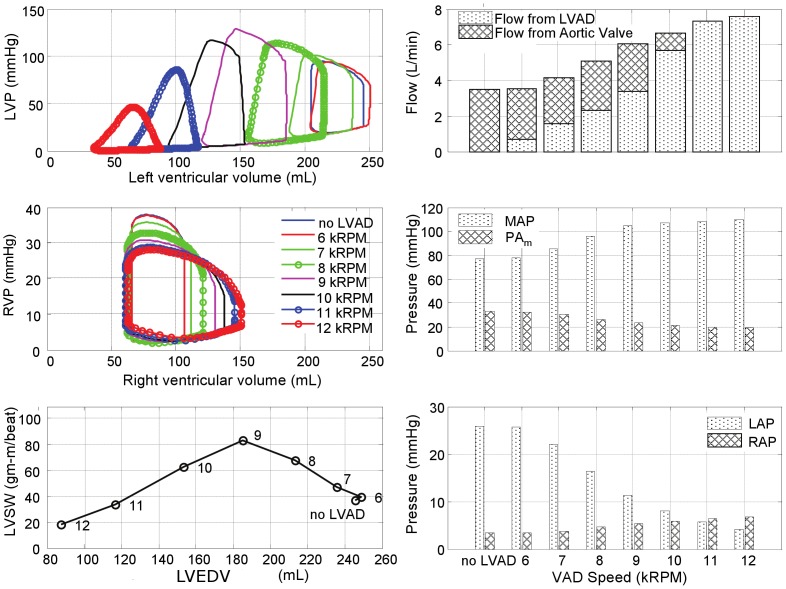
Simulated hemodynamics over a range of LVAD support (from 6k to 12k RPM) for the case of LV failure. With increased speed, the LV PV loops shift leftward, while the locus of maximal LV pressure exhibited unimodal response with a peak at 9k RPM, also reflected in LVSW. Total cardiac output however increased monotonically with LVAD speed, although the LV contribution decreased precipitously for speeds above 9k RPM. (LVP: left ventricular pressure; RVP: right ventricular pressure; LVSW: left ventricular stroke work; MAP: mean arterial pressure; PAm: mean pulmonary arterial pressure; LAP: mean left atrial pressure; RAP: mean right atrial pressure.)

**Figure 7:**
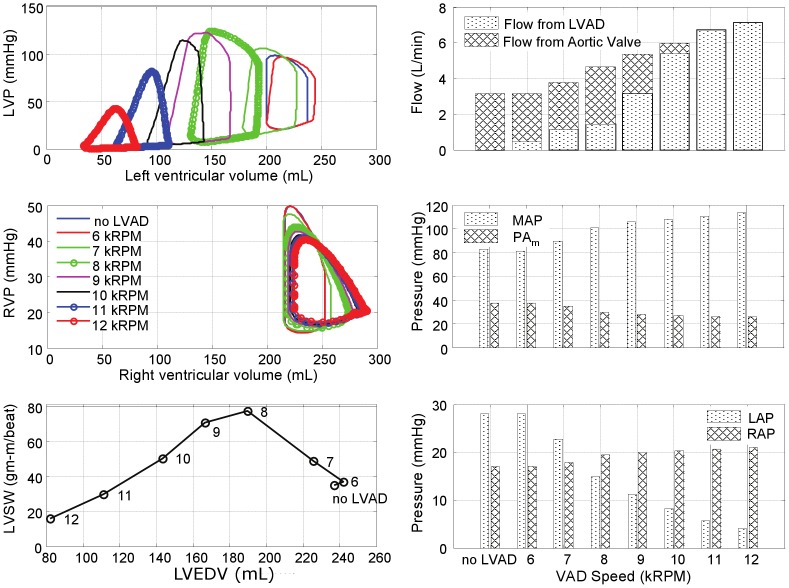
Simulated hemodynamics over a range of LVAD support (from 6k to 12k RPM) for the case of bi-ventricular failure. The peak LVSW occurred at a lower speed than the case of LVF alone (8k RPM versus 9k RPM.)

**Figure 8:**
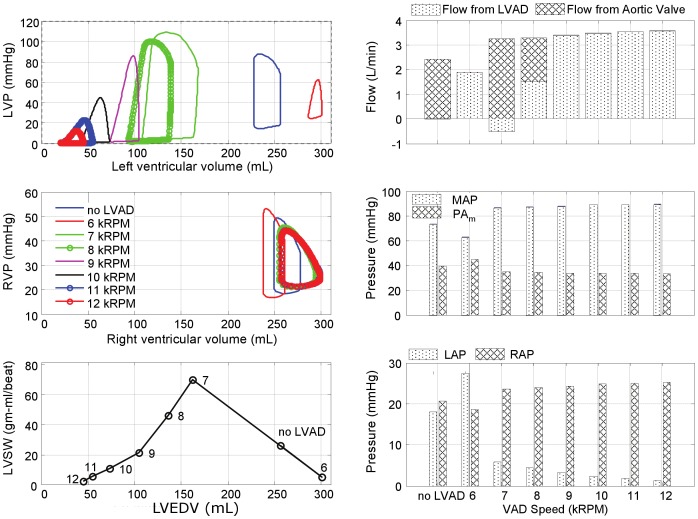
Simulated hemodynamics over a range of LVAD support (from 6k to 12k RPM) for the case of bi-ventricular failure with pulmonary hypertension.

**Figure 9:**
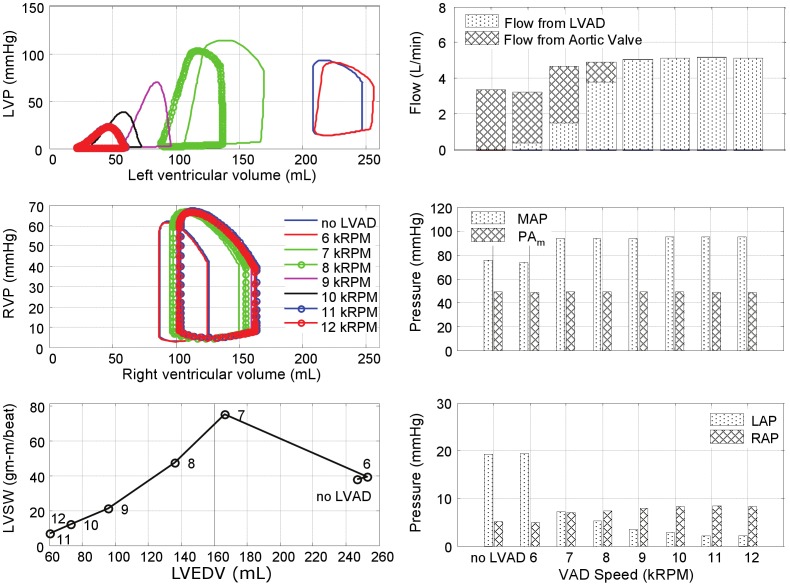
Simulated hemodynamics over a range of LVAD support (from 6k to 12k RPM) for the case of LV failure with pulmonary hypertension. The total CO reached a plateau at LVAD speed of 8k RPM.

The effects of LVAD support for each of the four pathological simulations are further illustrated in Fig. 6–9. In the LVF condition (Fig. 6), as the LVAD speed was increased from 6k to 12k RPM, the PV loops shifted leftward as the total CO increased from 3.5 to 7.6 L/min. The corresponding decrease in LV preload was rather dramatic: LAP from 26 mmHg to 4 mmHg and the end diastolic LV volume from 246 mL to 87 mL; while MAP increased from 77 to 110 mmHg. Concurrently, the RV preload (RAP) remained within the normal range, increasing slightly from 3 to 7 mmHg and afterload (PAm) reduced from 29 to 12 mmHg. The maximum LVSW (83 gm-m/beat) was however achieved at the intermediate speed of 9k RPM, providing 6.0 L/min total CO. At this optimal speed, the corresponding LAP was 11 mmHg, within the normal range and the maximum LV volume was 185 mL.

Fig. 7 provides the hemodynamic effects of LVAD in the Bi-F condition. In the absence of pulmonary hypertension, the total cardiac output was minimally affected by RV dysfunction, ranging from 3.2 to 7.2 L/min over the LVAD speed range of 6k–12k RPM. The effect of LVAD on the reduction of LAP and the maximum LV volume was similar to the LVF case: from 28 to 8 mmHg, and from 243 to 81 mL, respectively. Maximal LVSW (77 gm-m/beat) was achieved at the intermediate speed of 8k RPM, providing 4.7 L/min total CO. The optimal speed was however reduced to approximately 8k RPM, at which the corresponding LAP was 15 mmHg and the maximum LV volume was 191 mL. The presence of RVF resulted in an elevation of RAP (17 to 21 mmHg over the speed range) and a commensurate increase in the maximum RV volume between 252 and 290 mL. At the optimal speed, RAP and the maximum RV volume were 20 mmHg and 275 mL, respectively.

The introduction of moderate- to severe pulmonary hypertension to the condition of bi-ventricular failure (Fig. 8) caused a dramatic reduction in total cardiac output, which did not exceed 3.6 L/min despite maximal LVAD speed. At the lowest speed, 6k RPM, the total output was approximately 1.9 L/min, equivalent to the CO without the LVAD and at this speed the LV was distended into the overloaded region and incapable of generating sufficient pressure to open the aortic valve. A slight increase in speed to 7k RPM provided sufficient unloading to increase the LV contribution, however at this speed the pump output was inadequate to fully overcome arterial afterload resulting in net regurgitant flow through the LVAD. A further increase in LVAD speed to 8k RPM further unloaded the LV, reducing its contribution, which was compensated by a net positive flow through the LVAD, resulting in a total CO of 3.3 L/min. Above 8k RPM, the LV was sufficiently unloaded to eliminate aortic flow; yet the net CO reached a plateau at approximately 3.3–3.6 L/min, caused by concomitant RV overload (RAP
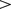
20 mmHg and the maximum RV volume
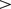
250 mL) which limited venous return to the left heart.

The hemodynamics were somewhat improved by reversing RVF; however pulmonary hypertension continued to limit the overall CO. (See Fig. 9.) Similar to the case of Bi-F with PVR+, the total CO reached a plateau (4.9 L/min) at 8k RPM LVAD speed, from which further increase of pump speed caused closure of the aortic valve. The maximum LVSW (75 gm-m/beat) was attained at 7k RPM. The PAm remained elevated at approximately 49 mmHg over the full range of LVAD speed. Although the immediate hemodynamic benefit of LVAD support was limited in this case, it is important to note the dramatic reduction of LAP which may, over time, reverse pulmonary congestion and reduce pulmonary vascular impedance and pulmonary hypertension.

### Comparison to Clinical Metrics of Cardiac Recovery

**Figure 10:**
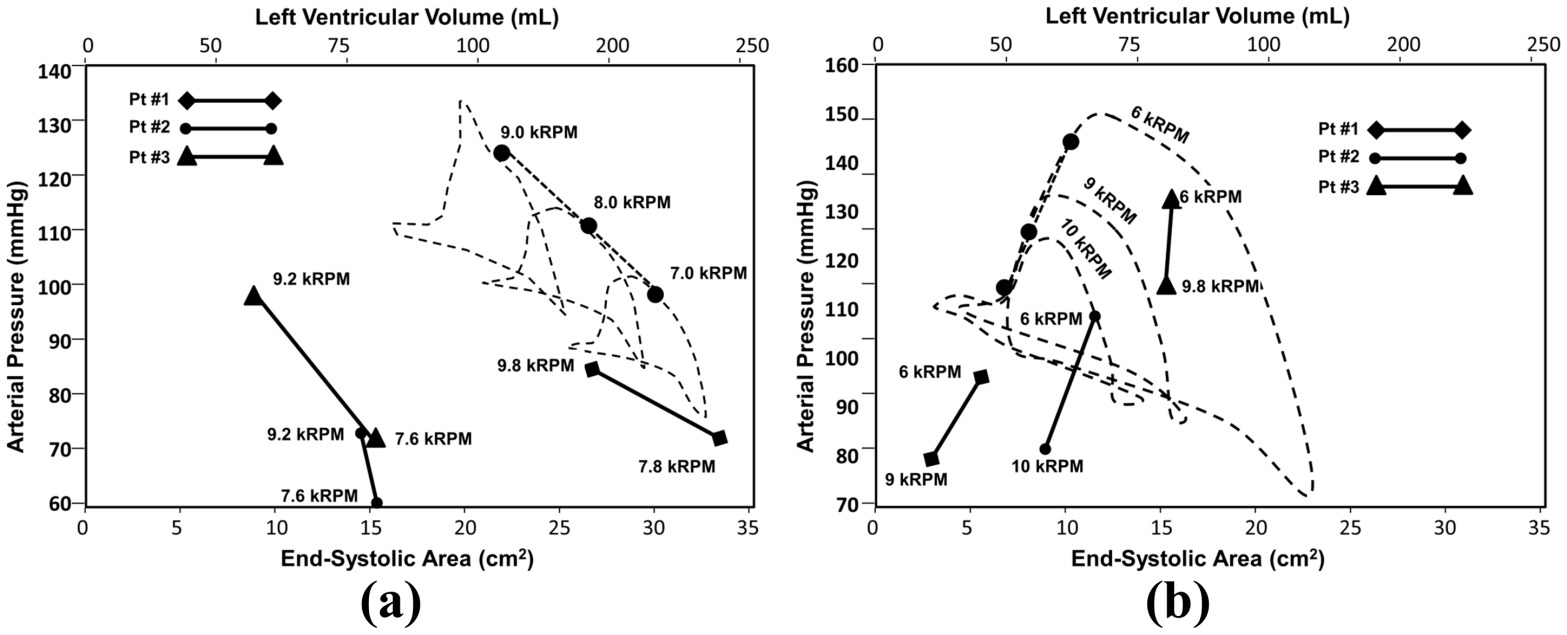
Comparison of clinical recording of end systolic arterial pressure (AP) vs. LV area (LVA) recorded by echocardiography and finger plethysmography (solid lines) with corresponding simulation (dashed lines.) (a) Patients who were deemed VAD dependent exhibit decrease of AP with decreased VAD support (increased preload); (b) whereas patients who were successfully weaned from LVAD support exhibited a physiological preload response: increasing AP with increased preload. The simulations (in which LVV was substituted for LVA) exhibited similar behavior.

Fig. 10 provides arterial pressure-volume loops from simulation (end systolic pressure-volume points labeled by black dots) combined with the locus of arterial end-systolic pressure and area from six patients enrolled in the UPMC ventricular recovery study: three of which were deemed LVAD-dependent (Fig. 10a) and three for whom the LVAD was successfully removed (Fig. 10b). For those patients dependent on LVAD, a decrease of LVAD speed resulted in enlargement of LV (reflected by increased end-systolic area: ESA) and compromised AP. Similarly, the simulation results for the case of LVF demonstrated that decreasing LVAD speed from extremal loading (9k RPM) lead to LV dilation and decrease of AP. Fig. 10b illustrates the corresponding simulation results for the normal (recovered) heart superimposed with clinical data of the 3 patient who were weaned from LVAD support. For these patients, a decrease of LVAD speed caused an increase in AP with only slight increase in ESA (less than 3 cm^2^). The simulation replicated this trend: as the LVAD speed was reduced from 10k to 6k RPM, the AP continuously increased with a slight increase of 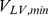. These results corroborate the underlying assumptions and hypothesis of the simulation model and thereby facilitate the identification of cardiac recovery.

## Discussion

Continuous-flow LVADs have recently proven their efficacy in providing long-term circulatory assistance, reversing symptoms of heart failure and, in certain cases, facilitating cardiac recovery [35], [36]. Nevertheless, strategies for prescribing and regulating the operating speed of cfLVADs are still ill-defined. This is particularly true in the context of cardiac recovery, in which ventricular re-loading may play an important therapeutic role [37].

This study was designed to investigate the relationship of level of mechanical circulatory support upon hemodynamics and ventricular energetics, in the context of dilated heart failure. The main findings provided by a lumped parameter simulation are: (1) a rightward shift of the pressure-volume (PV) loop, (2) a concomitent decrease in LVESP, (3) increase of LVEDP and LVEDV, and (4) in the most severe cases of cardiac overloaded, the ventricle exhibits a paradoxical response to preload wherein both LVESP and stroke volume decrease with increasing preload.

A novel feature of this model was the incorporation of a pathological ESPVR that represents the over-distended ventricle more accurately than a conventional, monotonically increasing function. The ventricular ESPVR has most commonly been regarded as linear, but in some cases has been represented by a quadratic function, flattening with high preload [38], [39]. In one such study, Burkhoff et al., working with isolated dog hearts, found that the curvilinearity of the ESPVR is dependent upon contractility [40]. By extrapolating the ESPVR with a descending limb, rather than an ever increasing function, the dilated ventricle exhibited a unimodal Starling curve: having a maximal point of preload reserve, beyond which incremental distention caused a decline in output. We explicitly elected to use a bilinear, rather than a parabolic, approximation so as to to parameterize the overloaded ventricle with three physically-meaningful values: the slope of the traditional ESPVR, an additional pathological descending ESPVR, and the transition point there between. Because the PV loops straddle the transition cusp its shape (sharp versus rounded) had no effect on the resulting PV loops.

Due to limiting effect of the EDPVR, the use of a monotonically increasing ESPVR will not necessarily cause unbounded cardiac output. However, a monotonic model of the overloaded, decompensated ventricle is clearly inadequate for characterizing the therapeutic effect of decompression.

It should be acknowledged that there are a variety of mechanisms that may be responsible for the descending portion of ESPVR. In the acutely overloaded heart, there is the possibility for energy starvation, leading to muscle exhaustion. [41] In the words of E.H. Starling, when dilatation exceeds the optimum length of the muscle fiber and the muscle has to contract at such a mechanical disadvantage the heart fails altogether. [10]. Modern studies of mechanical circulatory support also allow for the possibility that sarcomeres may become stretched beyond their Starling reserve [36], [48].

Mitral regurgitation is another likely condition presented in chronic dilatation due to the distortion of the mitral annulus. Reversal, or alleviation of volume overload, for example by diuresis, has been shown to *increase* stroke volume with decreasing preload. [42], [43]. This is also consistent with the observations of Gorcsan et al. who observed an increase in cardiac contraction with LVAD unloading. [44]. While our simulation model, in fact, includes parameters for mitral and tricuspid insufficiency, we consciously elected to omit this condition from the present study so as to independently reveal the importance of the descending limb. Furthermore, the addition of these parameters as independent variables would render the experimental matrix unwieldy.

Within this context, the current simulation studies introduced a concept of *extremal loading*. This was identified as the degree of VAD support for which LV stroke work is maximized. Above this level of support, the incremental improvement in net cardiac output is diminished, and eventually reaches an asymptote, constrained by venous return and/or RV output. Although impossible to prove here, it is reasonable to hypothesize that this represents a speed set-point for feedback control that is advantageous to myocardial rehabilitation. While it may be controversial whether physiologic ventricular loading or cardiac exercise promotes reverse remodeling, there exists sufficient clinical evidence that chronic *unloading* results in myocardial disuse atrophy [45]–[48]. A future simulation study should be conducted that focuses specifically on the variety of independent variables that may effect remodeling.

The obvious implication is that under certain circumstances it would be undesirable to operate the LVAD at full support. This may appear contrary to clinical intuition: to place an artificial constraint on cardiac output. On the other hand, these simulations demonstrated that the incremental gain in pump output above the optimal speed was counteracted by a diminution of cardiac contribution, resulting in a zero-sum gain. Furthermore, there is growing evidence of the benefit of allowing the aortic valve to open periodically [36], [49]. This can only be achieved if the level of LVAD support is restricted, so as to allow sufficient preload to the native heart.

A further clinical consideration that would favor less-than-maximal LVAD output is the potential for exacerbating RV dysfunction [13]. These simulations illustrated the delicate balance between RV preload, afterload, and septal decompression, particularly in the case of pulmonary hypertension. For example, in the simulated case of biventricular failure, the reduction in pulmonary pressure permitted the RV to respond favorably to increased demand. In the extreme case of biventricular disease combined with elevated PVR, LVAD support alone was insufficient for restoring adequate hemodynamics.

Inasmuch as the ESPVR function is not static, but dependent on time-varying factors such as the progression/reversal of disease, inotropic state, duration of volume overload, etc., the model presented here is intended to be illustrative rather than definitive. The chosen point of extremal loading was somewhat arbitrary, as it would depend on the body mass index (BMI) as well as etiology and extent of dilatation. Hence, any mechanical circulatory support strategy based on this function should be personalized to a given patient. And since the function is time-varying, there is a need for continuous feedback control.

This is however confounded by the unavailability of observable state variables (pressure, flow, volume, etc.) in the actual clinical setting. Therefore they must be inferred from readily measurable sources, such as the electrical signals from the VAD itself [5]. In addition, minimally invasive measurements may be obtained periodically to assess ventricular function, such as the echocardiographic protocol described by Gorcsan et al. [44]. By varying the degree of VAD support it becomes possible to assess preload response and thereby calibrate a simulation model, personalized for a patient.

Apart from feedback control, the simulation model affords the opportunity to perform in-silico interventions, such as vasoactive drugs, diuretics, correction of mitral regurgitation, insertion of a right ventricular assist device, etc. It is also hoped that this simulation model may be employed to provide greater confidence for manual adjustment of pump speed, which is commonly avoided for fear of ventricular suck down, regurgitant flow, or ventricular overload.

While the present model defines the point of overload in terms of end systolic volume, physiologically it would be more correct to define it in terms of a critical sarcomere stretch, hence end-diastolic volume. A model based on this premise would not be readily compatible with the mathematical formulation here, but could possibly be implemented in terms of a unimodal SWI-EDV model. However, resulting hemodynamic response to LVAD support would be equivalent; inasmuch as the latter relationship is defined implicitly, rather than explicitly.

An additional limitation of the present model is omission of pathological vascular regulation that is common to end-stage heart failure. In lieu of an established rennin-angiotensin- aldosterone model, these regulatory effects could be introduced manually into the current baroreflex model in a future extension of this work. An additional aspect of cardiac failure that would be interesting to incorporate into a future study would be intra-ventricular dyssynchrony.

This study made a best effort to assure the accuracy of the simulation model presented here by reference to various data sources from the literature. However, definitive validation will require data from actual patients receiving cfLVAD support that exhibit a spectrum of heart failure. This is the goal of an ongoing clinical study by our group. Until such corroborating data are available, the present study serves as a prototypical template upon which a clinical study may be designed.

The future direction of this research will be to also develop extremum-tracking controllers [14], [15] that account for the observed unimodal response of the LV to unloading, and specifically to seek the optimal degree of unloading to guide cfLVAD therapy.

## Supporting Information

Appendix S1 Supporting material: Numerical representation of the left and right ventricles, septum, systemic and pulmonary vascular impedances; Kirchoffs set of thirteen state equations derived for the full cardiovascular model; Tables AI-AIV contains the parameter values and hemodynamic variables derived from the literature and used in the simulator under normal and different pathological conditions.
(TIF)
