# Supplementary figures and images for: Simulation of Dilated Heart Failure with Continuous Flow Circulatory Support
Source: PLoS One. 2014 Jan 17;9(1):e85234. doi: 10.1371/journal.pone.0085234 (PMC3894974; doi:10.1371/journal.pone.0085234)

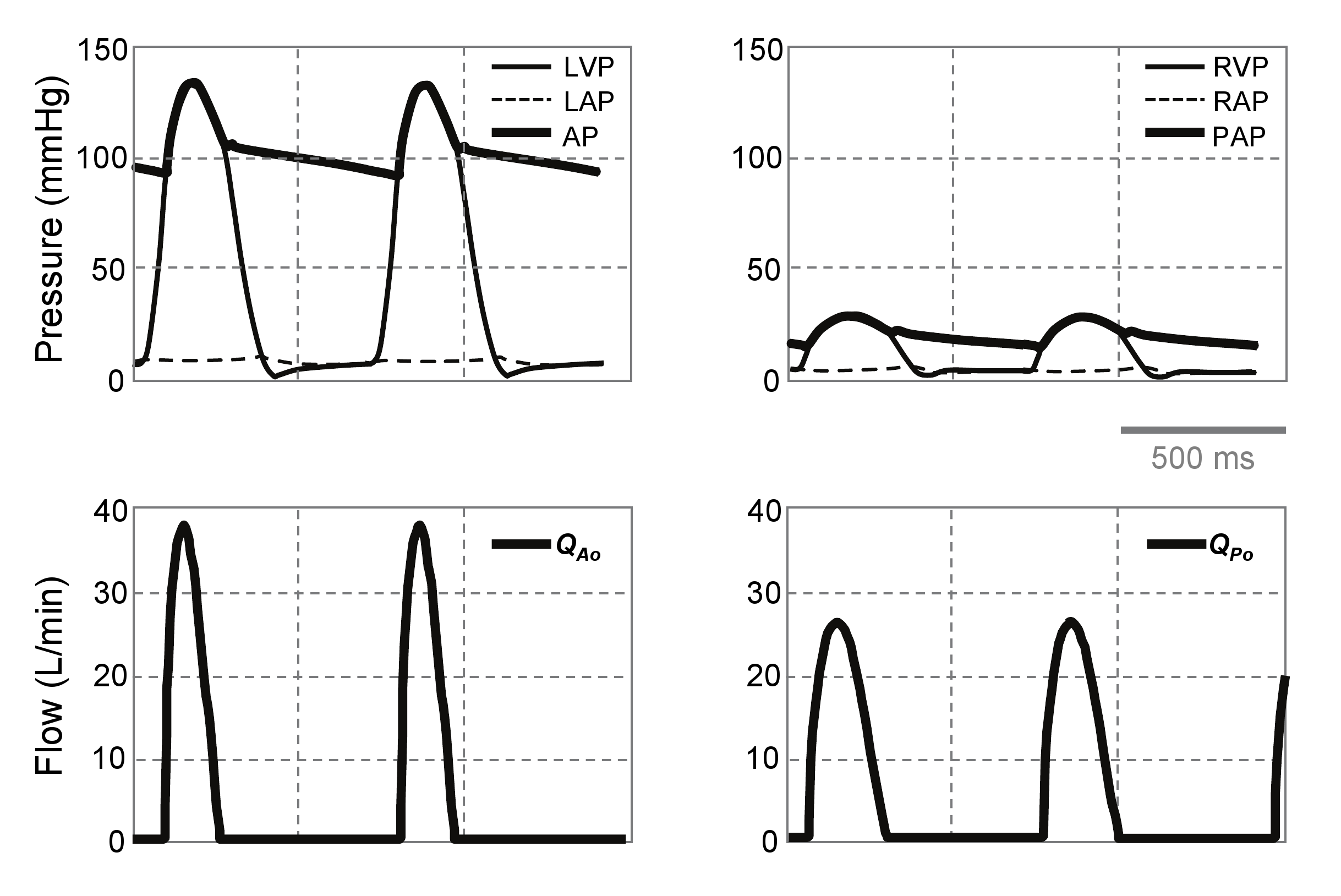

Supplement: Appendix S1 — Supporting material: Numerical representation of the left and right ventricles, septum, systemic and pulmonary vascular impedances; Kirchoffs set of thirteen state equations derived for the full cardiovascular model; Tables AI-AIV contains the parameter values and hemodynamic variables derived from the literature and used in the simulator under normal and different pathological conditions. (TIF) [file pone.0085234.s001.tif]
